# Y-SNPs Do Not Indicate Hybridisation between European Aurochs and Domestic Cattle

**DOI:** 10.1371/journal.pone.0003418

**Published:** 2008-10-14

**Authors:** Ruth Bollongino, Julia Elsner, Jean-Denis Vigne, Joachim Burger

**Affiliations:** 1 Palaeogenetics Group, Institute of Anthropology, Johannes Gutenberg University Mainz, Mainz, Germany; 2 CNRS, Muséum national d́Histoire Naturelle, Département Ecologie et gestion de la Biodiversité, USM 303, Archéozoologie, CP 56, Paris, France; University College London, United Kingdom

## Abstract

**Background:**

Previous genetic studies of modern and ancient mitochondrial DNA have confirmed the Near Eastern origin of early European domestic cattle. However, these studies were not able to test whether hybridisation with male aurochs occurred post-domestication. To address this issue, Götherström and colleagues (2005) investigated the frequencies of two Y-chromosomal haplotypes in extant bulls. They found a significant influence of wild aurochs males on domestic populations thus challenging the common view on early domestication and Neolithic stock-rearing. To test their hypothesis, we applied these Y-markers on Neolithic bone specimens from various European archaeological sites.

**Methods and Findings:**

Here, we have analysed the ancient DNA of 59 Neolithic skeletal samples. After initial molecular sexing, two segregating Y-SNPs were identified in 13 bulls. Strikingly, our results do not support the hypothesis that these markers distinguish European aurochs from domesticated cattle.

**Conclusions:**

The model of a rapid introduction of domestic cattle into Central Europe without significant crossbreeding with local wild cattle remains unchallenged.

## Introduction

**Figure 1:**
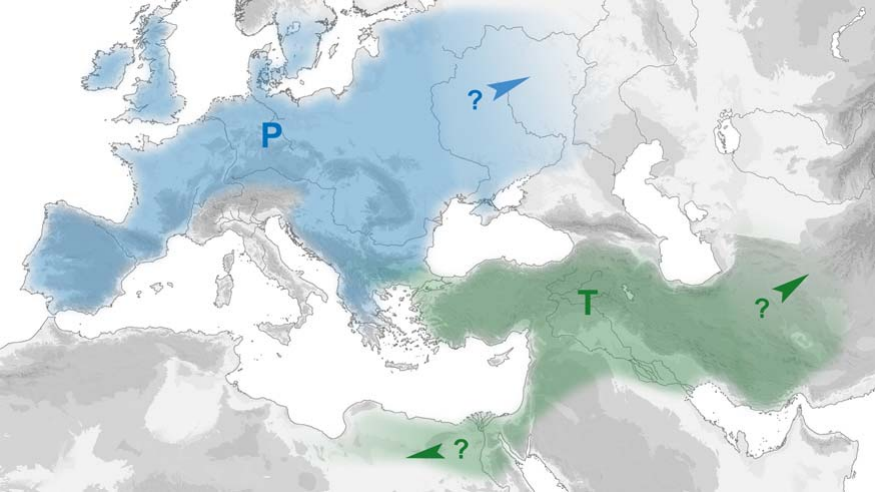
Assumed distribution of aurochs mt-haplogroups in Western Eurasia. P (blue) = *primigenius* haplogroup of European aurochs populations, T (green) = taurine haplogroups (T, T1, T2, T3) of Near and Middle Eastern aurochs populations. The arrows indicate areas where no aurochs samples have been investigated so far. Furthermore, it is not yet clear whether T-types also appeared in wild populations of Thrace and the Balkans.

Molecular genetic analyses of prehistoric bovines revealed that European taurine domestic cattle (*Bos taurus*) originate from a Near Eastern population of the wild ox or aurochs (*Bos primigenius*). The mitochondrial lineages of Near Eastern aurochs and their domestic descendants belong to the T-haplogroups (T = *taurus*, subdivided into T, T1–T3). The European aurochs, which belongs to a different population (haplogroup P, see figure 1), became extinct without any traceable genetic contribution to the domestic herds [1], [2], [3].

However, the majority of molecular genetic studies focused on matrilinear inherited mitochondrial DNA (mtDNA), thus solely reflecting the history of female individuals. Thus the lack of mitochondrial P-lineages in extant European cattle actually only indicates that female aurochs were not included into domestic populations. Without additional analyses of Y-chromosomal patrilines, male introgression cannot be ruled out. The question of whether and to what extent crossbreeding occurred in Europe has been the subject of much debate. Its proponents argue that early farmers often did not keep their cows separated from wild oxen and that crossbreeding might have been used intentionally to improve the breeding stock and increase their numbers. Alternatively, some researchers have argued that hybrids could have been difficult to handle and, based on historical accounts, that farmers would have killed aurochs bulls who mate with domestic cows, as the cow will “either miscarry, or give birth to non-viable young” [4].

Domestic cattle are significantly smaller than their wild progenitors, thus one could assume that hybridisation is easily detectable by morphological and osteometric means. But the interpretation of intermediate-sized bones is impeded by the pronounced sexual dimorphism of bovines. This results in a broad overlap in the body-size variation of aurochs and cattle, meaning that a medium-scaled bone can either belong to a female aurochs or to a domestic bull.

Crossbreeding between domestic bulls and female aurochs is highly unlikely to be detected by molecular genetic means, as the offspring would remain in the wild population and not in the settlements. But offspring of aurochs bulls and domestic cows would be raised within the domestic herds. Nevertheless, they would still remain undetected through mtDNA haplotyping.

Ancient DNA studies on Y-chromosomes are comparatively rare because: (i) the preservation of nuclear DNA (ncDNA) is far worse than for mtDNA, (ii) patrilines are less intensively studied and segregating sites are not well known, (iii) only males carry a Y-chromosome, thus approximately only 50% of the samples are suitable for analysis but the morphological identification of bones from male individuals is often impossible.

**Figure 2:**
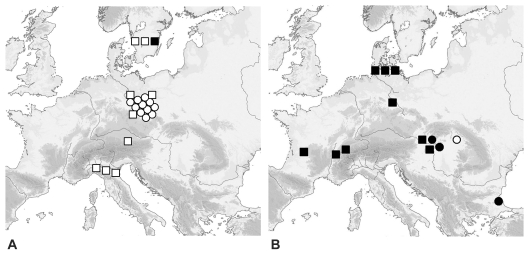
Y-chromosome data of ancient samples from: A) Götherström et al. (2005) and B) this study. White = Y1, black = Y2, squares = European aurochs mt-type (P), circles = Near Eastern domestic mt-type (T).

Consequently, Götherström and colleagues [5] sequenced 3.5 kb from the Y-chromosome genes *DB*Y, *UBE1Y*, *UTY*, and *ZFY* from 180 modern samples. They found two segregating sites (see table 1), together characterising two haplotypes (Y1 and Y2). Within European contemporary cattle, both haplotypes are prevalent, with Y1 being more frequent in north-western Europe, whereas Y2 is more dominant in southern Europe. As Y2 is the only haplotype found in Anatolian breeds, the authors suggest that it represents the domestic taurine cattle population originating from the Near East, whereas Y1 might reflect the European aurochs haplotype. This hypothesis was further tested by typing 21 ancient samples (11 aurochs, 4 domestic cattle and 5 intermediate size, and 1 unknown, see table 2 and figure 2), all of which belonged to the Y1 haplotype, except one Swedish auroch. This supported the authors' assumption that European aurochs belong to the Y1-haplogroup.

**Table 1:** Segregating sites according to Götherström *et al.* (2005).

Haplotype	Marker and polymorphism	
	*UTY19*	*ZFY5 indel*
Y1	G	Deletion
Y2	T	GT-insertion

Notes: *UTY19*: position 423 in Genbank accession AY936543; *ZFY5 indel*: position 698 and 699 in Genbank accession AF241271.

**Table 2:** Results of the Y-SNP analysis of 13 Meso- and Neolithic wild and domestic bulls compared to the haplotypes of 21 ancient samples from Götherström *et al.* 2005.

Sample	Site/Country	Phenotype	mt-haplotype	Y-haplotype
**This study**:				
ALB4	Albertfalva, Hungary	BP	**P**	**Y2**
CAT1	Cave à ĺOurs, France	BP	**P**	**Y2**
HAL1	Halle, Germany	BP	**P**	**Y2**
PAR1	Grotte du Gardon, France	BP	**P**	**Y2**
ROS3	Rosenhof, Germany	BP	**P**	**Y2**
ROS5	Rosenhof, Germany	BP	**P**	**Y2**
ROS7	Rosenhof, Germany	BP	**P**	**Y2**
ROU6	Roucadour, France	BP	**P**	**Y2**
SVO 3	Svodin, Slovakia	BT	**P**	**Y2**
ALB3	Albertfalva, Hungary	BP?	T3	Y2
POL5	Polgár-Csöszhalom, Hungary	B sp.	**T3**	**Y1**
AP7	Asagi Pinar, Turkey	BT?	T	Y2
SVO1	Svodin, Slovakia	BT	T3	Y2
**Götherström et al. 2005:**				
Lzz3287	Sweden	BP ***	**-**	Y1
Lzz3348	Sweden	BP ***	**-**	**Y2**
Lzz3343	Sweden	BP ***	**-**	Y1
2M3886	Italy	BP ***	**-**	Y1
3M3884	Italy	BP ***	**-**	Y1
4	Italy	BP ***	**-**	Y1
DD10	Germany	BP ***	**-**	Y1
DD23	Germany	BP ***	**-**	Y1
DD56	Germany	BP ***	**-**	Y1
Aut10:2	Austria	BP ***	**-**	Y1
DD73	Germany	BP ***	**-**	Y1
DD35	Germany	B sp. **	**-**	Y1
DD24	Germany	B sp. **	**-**	Y1
DD25	Germany	B sp. **	**-**	Y1
DD27	Germany	B sp. **	**-**	Y1
DD21	Germany	B sp. **	**-**	Y1
DD29	Germany	BT *	**-**	**Y1**
DD39	Germany	BT *	**-**	**Y1**
DD61	Germany	BT *	**-**	**Y1**
DD64	Germany	BT *	**-**	**Y1**
DD22	Germany	B. sp.	**-**	**Y1**

Notes: BP = *Bos primigenius* (aurochs), BT = *Bos taurus* (domestic cattle), B sp. = *Bos*, not further determinable, ? = insecure determination. mt-haplotypes: P = aurochs, T3/T = domestic cattle. Haplotypes in bold mark samples, where mt- and Y-haplotypes would identify hybrids according to Götherström and colleagues. ^***^ = *Bos primigenius*, ^**^ = intermediate size, ^*^ = *Bos taurus*.

However, these conclusions are based largely on the modern haplotype distribution which might reflect recent breeding practices rather than prehistoric herd management. Thus, we investigated the haplotype defining Y-SNPs in aDNAs from Neolithic bone samples with known mtDNA haplotypes originating from archaeological sites in Central, Western, and South-Eastern Europe.

## Results

No contaminations could be observed in either the extraction or PCR negative controls, nor could we observe mixed haplotyes during sequence replication. Twenty-nine out of the 59 samples yielded replicable nuclear DNA. No inconsistent haplotypes were observed. Sixteen were identified as females, 13 as males (see table S1 for sample details and table S4 for Genbank accession numbers in the electronic supporting information (SI)). Y-chromosomal SNPs were detected in all 13 bulls (see table 2). The results of these ancient samples confirm that both segregating sites together form the two haplotypes, as previously found through analysis of modern DNAs (e.g. a G allel at UTY19 is always connected with a deletion in the ZFY5 locus). The 13 bulls stem from nine different sites spread over five countries (Hungary, Turkish Thrace, France, Germany and Slovakia).

Most of the mitochondrial P or T-types correspond to the morphological and/or chronological assignment to either the *primigenius* population or the *taurus* population, except two: ALB3 was labelled as “determination insecure” and SVO3 was described as “small”; but bone fragments of both samples were not measurable. The mitochondrial lineages of nine samples belong to the P haplogroup of European aurochs, four samples carry the T/T3-haplogroup that is found in domestic animals (see figure 2). The Y1 haplotype was only found in one sample (with a T3 mt-type), all other samples showed the ‘Near Eastern’ Y2 type (see figure 2 and table 2). Comparing the mt and Y-haplotypes, 10 out of 13 bulls would be hybrids, according to Götherström and colleagues.

## Discussion

None of the 150 sequences obtained in previous studies of ancient samples [2], [3], [7] produced a T-lineage with definite aurochs specimens (determined either by their pre-Neolithic date or morphology). Neither, a P-haplotype ever came from a doubtless domestic cattle bone. The analysis of mitochondrial DNA therefore is an appropriate tool to discriminate European aurochs populations from taurine populations of western Asia. The morphological determination of the specimens in this study is consistent with their assignment to one of the two populations. One exception, ALB3, was tentatively assigned to aurochs based on morphological criteria, although marked as “insecure” and it revealed a mitochondrial T-haplotype. The size variation of domestic cattle and aurochs overlaps significantly, leaving intermediate-sized bones like ALB3 unidentifiable, especially when the bone fragment is not measurable and the sex unknown. The fact that ALB3 is male explains the large size of this bone and its assignment to *Bos primigenius*.

SVO3 is a special case, as this is the only specimen that is morphologically attributed as *Bos taurus* due to its “small” size, but revealed a *primigenius* mt-lineage. As this individual was identified as a male, it should have been easily distinguishable from domestic cattle. Thus ambiguous assignment of SVO3 may indicate that it is a hybrid. Interestingly, according to the Y-SNPs criteria of Götherström and colleagues, this sample is one of three specimens that should not be considered as a hybrid.

It is also striking that all the bulls, except SVO3, would be interpreted as hybrids. In this case, male introgression occurred in both directions: domestic bulls breeding with aurochs cows (ALB4, CAT1, HAL1, PAR1, ROS3, ROS5, ROS7, ROU6 and SVO3) and aurochs bulls breeding with domestic cows (POL5). ALB3, AP7 and SVO1 would be regarded as “pure” imported domestic cattle. In other words, out of four domestic cattle, only one would be a hybrid, whereas all of the nine aurochs would be defined as hybrids. This is highly unlikely. There are two possibilities to explain the nine individuals that have an aurochs matrilineage and a taurine patrilineage: 1) They are domesticated animals. In this case, female aurochs were introduced into the domestic herds where they mated with taurine bulls and their offspring remained in the settlement. 2) They are feral, i.e. domestic bulls mate with free-ranging aurochs cows and their offspring remain in the wild population. The first possibility can be disregarded because the complete loss of P-lineages in extant European cattle populations precludes the widespread introduction of female aurochs into domestic herds. Thus, the second explanation remains the more likely. But, as the specimens were recovered from settlements, those wild hybrids must have been hunted by coincidence and brought into the settlement to be retrieved by archaeologists. It is not convincing that this was the case for all aurochs samples. But above all, it is inconceivable that all aurochs samples should stem from hybrids, though crossbreeding is expected to be a rather rare event. In summary, there is no plausible way to interpret the results as indicators for crossbreeding. A more parsimonious explanation would be that European aurochs were comprised of both Y-haplotypes without any impact on Near Eastern cattle or their descendants. Our data suggest that the two haplogroups do not have a separate geographic origin and thus do not distinguish European and Near Eastern lineages. Most strikingly, one of the Swedish aurochs samples [5] produced an Y2 haplotype (see table 2). It is noteworthy that the aurochs in Sweden became extinct before the first arrival of domestic cattle, thus this example shows that Y2 is prevalent in European wild populations prior to the arrival of domesticates. Additionally, if the geographic separation of the lineages held, it would show that the extremely high percentage of Y1 in contemporary Swedish cattle does not reflect widespread hybridisation, but rather genetic drift or bottleneck that resulted from the ancestral population outside of Sweden.

How can the different results between our study and the one of Götherström et al. [5] be explained? We analysed ancient samples from a broad geographic region, whereas the majority of the ancient samples in the study by Götherström et al. originate from a small region in eastern Germany and might reflect a reduced local diversity. Unfortunately, no information about the mitochondrial lineage of the samples, which could have complemented the morphological identification, was given by Götherström et al.. Moreover, eleven of their ancient samples with intermediate size or sure domestic provenance belonged to the Y1 haplogroup. Unfortunately, the authors give no explanation for the missing Near Eastern haplotype amongst the ancient domestic specimens (except in one Swedish aurochs, see below and table 2). Thus, the suggestion that Y1 represents European aurochs and Y2 descendants of Near Eastern cattle is mainly based on the modern haplotype distribution and might be biased by historic breeding practices and genetic drift.

In conclusion, the ancient distribution of the Y1 and Y2 haplotypes suggests that they do not discriminate European and Near Eastern Y-chromosomal lineages. As a consequence, there is still no patrilinear marker for investigating possible male introgression between imported cattle and European aurochs. So far, the importation of taurine cattle from its Near Eastern centre of domestication into Europe without subsequent hybridisation with local wild cattle populations remains the preferred model for the origin of European cattle.

## Materials and Methods

The analyses were conducted on 59 samples of domestic cattle and aurochs from different geographic regions in Europe (see table S1 in SI for details). The aurochs specimens from Rosenhof are late Mesolithic and thus predate the domestication; all other bones are classified according to their size.

All of these samples had feasibly amplified mtDNA in previous studies [2], [3], [7] and additional unpublished data. Molecular sexing was carried out (see below) in order to assess ncDNA preservation and to identify male individuals.

Samples were processed in a laboratory that is solely dedicated to ancient DNA, following strict international standards. Phenol-chloroform extraction and PCR were conducted as described in Burger et al. [6]. PCR reagents and concentrations are given in table S3 in SI. Sex-specific primers for the ZFX/Y gene were used for sex determination of the samples (see table S2 ESM).

Within all identified bulls, two markers (UTY19 and ZFY5 ind) as described by Götherström et al. [5] were amplified, but newly-designed primer sets were used (see table S2 in SI). The results were replicated at least three times for each marker (from two independent extractions). PCR products of both segregating sites were directly sequenced with an Applied Biosystems 3130 Genetic Analyser. Sequences were aligned with MegAlign and SeqMan of the Lasergene software package.

## Supporting Information

Table S1 Sample details
(0.25 MB DOC)

Table S2 Primers
(0.04 MB DOC)

Table S3 PCR protocol
(0.03 MB DOC)

Table S4 GenBank accession numbers of sequences presented in this study.
(0.04 MB DOC)
