# Supplementary material for: Y-SNPs Do Not Indicate Hybridisation between European Aurochs and Domestic Cattle
Source: PLoS One. 2008 Oct 14;3(10):e3418. doi: 10.1371/journal.pone.0003418 (PMC2561061; doi:10.1371/journal.pone.0003418)
Supplement: Table S1 — Sample details (0.25 MB DOC) [file pone.0003418.s001.doc]

Table S1: Sample details. F= female, (F)= probably female, M= male, (M)= probably male, n.a.= not amplifiable. A, B, C= number of extractions; 1, 2, 3=number of PCR products. Hpt = haplotype. Males used in this study are highlighted in grey.

| Code | Site | Archaeolog. code | Date | Sourced by | Pheno-  type | mt-hpt | Gen. sex | Amplification success  ZFX3/ZFY3 | | | Y-hpt | Amplification success  YSNP1  YSNP2 | | |
| --- | --- | --- | --- | --- | --- | --- | --- | --- | --- | --- | --- | --- | --- | --- |
| Alb1 | Albertfalva, Hungary | 65 obj 777 (small) | 2500 BC, Bell Beaker | A Choyke | *Bos sp.* | T3 | F | A/2 | B/2 |  |  |  |  |  |
| Alb2 | Albertfalva, Hungary | 65 obj 777 (big) | 2500 BC, Bell Beaker | A Choyke | *B. p.* | P | n. a. |  |  |  |  |  |  |  |
| Alb3 | Albertfalva, Hungary | 296 obj 3115 | 2500 BC, Bell Beaker | A Choyke | *B. p.?* | T3 | M | A/2 | B/2 |  | Y2 | A/2 | B/2 |  |
|  |  |  |  |  |  |  |  | A/2 | B/1 |  |  | A/3 | B/1 |  |
| Alb4 | Albertfalva, Hungary | 62 obj 1246 | 2500 BC, Bell Beaker | A Choyke | *B. p.* | P | M | A/2 | B/2 |  | Y2 | A/2 | B/1 |  |
|  |  |  |  |  |  |  |  | A/2 | B/1 |  |  | A/3 |  |  |
| Ap7 | Asagi Pınar, Turkey | 26-AP ´99 9R 136.- | 5250-5080 BC cal, Karanovo | H Hongo, M Özdogan | *B. t.?* | T | M | A/2 | B/2 |  | Y2 | A/2 | B/2 |  |
|  |  |  |  |  |  |  |  | A/2 | B/2 |  |  | A/2 | B/2 |  |
| Ber6 | Berettyószentmárton Hungary | 56.11.979 | Neolithic | I Vörös | *Bos sp.* | T3 | F | A/2 | B/2 |  |  |  |  |  |
| Berc2 | Bercy, France | T-BER/02 | Chasséen | A Tresset | *Bos sp.* | T3 | n. a. |  |  |  |  |  |  |  |
| Berc3 | Bercy, France | T-BER/03 | Chasséen | A Tresset | *B. t.* | T3 | (F) | A/1 | B/1 |  |  |  |  |  |
| Bo1 | Baume d`Oulen, France | V-BO-1 | Cardial | J-D Vigne | *Bos t.* | T3 | (F) | A/2 |  |  |  |  |  |  |
| Bo3 | Baume d`Oulen, France | V-BO-3 | Epicardial | J-D Vigne | *Bos t.* | T3 | (F) | A/2 |  |  |  |  |  |  |
| Bor1 | Bordusani Popina, Roumania | RO BORD A6 | Gumelnita-Karanovo | A Balasescu | *B. t.* | T3 | (F) | A/2 |  |  |  |  |  |  |
| Bor2 | Bordusani Popina, Roumania | RO BORD A4 | Gumelnita-Karanovo | A Balasescu | *B. t.* | T3 | F | A/2 | B/1 |  |  |  |  |  |
| Cad1 | Chateau d`Oex, Switzerland | - | ca. 3000 BP | L Chaix | *Bos sp.* | T3 | (F) | A/2 |  |  |  |  |  |  |
| Cat1 | Cave à l´Ours, France | CP/33.116 | 3694 BC cal | L Chaix | *B. p.* | P | M | A/2 | B/2 |  | Y2 | A/2 | B/1 | C/1 |
|  |  |  |  |  |  |  |  | A/2 | B/1 | C/1 |  | A/2 | B/2 |  |
| Che1 | Cheia, Roumania | RO CHE A7 | Hamangia | A Balasescu | *B. t.* | T3 | n. a. |  |  |  |  |  |  |  |
| Der1 | Derenburg-Steinkuhlenberg, Germany | HK 87: 183i | Bernburg, 3600 BC | H-J Döhle | *B. t.* | T3 | F | A/2 | B/1 |  |  |  |  |  |
| Eil1 | Eilsleben, Germany | HK 83: 1040l | 5000 BC, LBK | H-J Döhle | *B. t.* | T3 | n. a. |  |  |  |  |  |  |  |
| Eil6 | Eilsleben, Germany | HK 83: 702i | 5000 BC, LBK | H-J Döhle | *B. p.* | P | (F) | A/2 |  |  |  |  |  |  |
| Eil9 | Eilsleben, Germany | HK 85: 138p | 5000 BC, LBK | H-J Döhle | *B. t.* | T3 | F | A/2 | B/2 |  |  |  |  |  |
| God3 | Goddelau, Germany | GO 73i-1 | oldest LBK | H-P Uerpmann | *Bos sp.* | T3 | n. a. |  |  |  |  |  |  |  |
| Hal1 | Halle, Germany | 97 A SF48 | 100 BC, Latène | H-J Döhle | *Bos sp.* | P | M | A/2 | B/2 |  | Y2 | A/2 | B/1 | C/1 |
|  |  |  |  |  |  |  |  | A/2 | B/2 |  |  | A/2 | B/1 | C/1 |
| Lju1 | Mala Triglavca, Slovenia | ? | (late)Neolithic | M Budja | *B. t.* | T3 | F | A/2 | B/1 |  |  |  |  |  |
| Lju3 | Mala Triglavca, Slovenia | ? | (late)Neolithic | M Budja | *Bos sp.* | P | n. a. |  |  |  |  |  |  |  |
| Nes2 | Neustadt (Holstein), Germany | LA 156/04  N 100-101  E 116-117 | 4500-4100 cal BC | S Hartz, U Schmölke | *B. t.?* | P | (F) | A/2 |  |  |  |  |  |  |
| Nmr22 | Nieder-Mörlen, Germany | 4/2. 1162/25890 EV98/2 | Flomborn | S Schade-Lindig | *B. t.?* | T3 | F | A/2 | B/2 |  |  |  |  |  |
| Nmr24 | Nieder-Mörlen, Germany | 7/1. 877/25191 | Flomborn |  | *B. t.* | T3 | F | A/2 | B/2 |  |  |  |  |  |
| Par1 | Grotte du Gardon, France | G90.K23:d44:22 | 3340-3150 BC cal | H-J Döhle | *B. p.* | P | M | A/2 | B/1 | C/1 | Y2 | A/2 | B/1 | C/1 |
|  |  |  |  |  |  |  |  | A/2 | B/1 | C/1 |  | A/2 | B/2 |  |
| Pol2 | Polgár-Csöszhalom, Hungary | 60.9.197 | Late-Neolithic | I Vörös | *Bos sp.* | T | (F) | A/2 |  |  |  |  |  |  |
| Pol5 | Polgár-Csöszhalom, Hungary | 60.9.1879 | Late-Neolithic | I Vörös | *Bos sp.* | T3 | M | A/2 | B/2 |  | Y1 | A/2 | B/1 | C/1 |
|  |  |  |  |  |  |  |  | A/2 | B/2 |  |  | A/2 | B/2 |  |
| Que1 | Quenstedt, Germany | 77:193 | BZ | H-J Döhle | *B. t.* | T3 | (F) | A/2 |  |  |  |  |  |  |
| Que3 | Quenstedt, Germany | 77:200 | BZ | H-J Döhle | *B. t.* | T | n. a. |  |  |  |  |  |  |  |
| Ros1 | Rosenhof, Germany | Ros 74 VI 148i (Altgrabung) | 4840 +/- 80 cal BC  4740 +/- 60 cal BC | S Hartz, U Schmölke | *B. t./ B. p.?* | P | F | A/2 | B/2 |  |  |  |  |  |
| Ros2 | Rosenhof, Germany | S 95,5-96  O 113,5-114  T -4,21 | End-Mesolithic | S Hartz, U Schmölke | *B. p.* | P | F | A/2 | B/1 |  |  |  |  |  |
| Ros3 | Rosenhof, Germany | S112,68  O 168,21  T -4,25 | End-Mesolithic | S Hartz, U Schmölke | *B. p.* | P | M | A/2 | B/2 |  | Y2 | A/2 | B/2 |  |
|  |  |  |  |  |  |  |  | A/2 | B/2 |  |  | A/1 | B/1 | C/1 |
| Ros5 | Rosenhof, Germany | Suchschnitt 2001  S 8-18  O 108-110  T -3,50-4,50 | End-Mesolithic | S Hartz, U Schmölke | *B. p.* | P | M | A/2 | B/2 |  | Y2 | A/2 | B/2 |  |
|  |  |  |  |  |  |  |  | A/2 | B/2 |  |  | A/2 | B/2 |  |
| Ros7 | Rosenhof, Germany | Suchschnitt 2001  S 8-18  O 108-110  T -3,50-4,50 | End-Mesolithic | S Hartz, U Schmölke | *B. p.* | P | M | A/2 | B/2 |  | Y2 | A/2 | B/2 |  |
|  |  |  |  |  |  |  |  | A/2 | B/2 |  |  | A/2 |  |  |
| Ros9 | Rosenhof, Germany | Ros 80 XIX 337 h (Altgrabung) | 4000 +/- 50 cal BC | S Hartz, U Schmölke | *B. t./ B. p.?* | T3 | F | A/2 | B/2 |  |  |  |  |  |
| Ros10 | Rosenhof, Germany | Ros 73 Ia 44h (Altgrabung) | End-Meso-/early Neolithic | S Hartz, U Schmölke | *B. t./ B. p.?* | P | F | A/2 | B/2 |  |  |  |  |  |
| Ros11 | Rosenhof, Germany | Ros 75 VIII 191g (Altgrab.) | End-Meso-/early Neolithic | S Hartz, U Schmölke | *B. t./ B. p.?* | P | F | A/2 | B/2 |  |  |  |  |  |
| Ros12 | Rosenhof, Germany | Ros 80 XIX 350 i (Altgrabung) | End-Meso-/early Neolithic | S Hartz, U Schmölke | *B. t./ B. p.?* | P | F | A/2 | B/2 |  |  |  |  |  |
| Rou1 | Roucadour, France | L-ROU/01 | Middle Neolithic, Chasséen C2a | J-D Vigne | *B. p.* | P | n. a. |  |  |  |  |  |  |  |
| Rou2 | Roucadour, France | L-ROU/02 | Middle Neolithic, Chasséen C2b | J-D Vigne | *B. p.* | P | n. a. |  |  |  |  |  |  |  |
| Rou3 | Roucadour, France | L-ROU/03 | Middle  Neolithic, Chasséen C2b | J-D Vigne | *B. p.* | P | n. a. |  |  |  |  |  |  |  |
| Rou4 | Roucadour, France | L-ROU/04 | Middle  Neolithic, Chasséen C2b | J-D Vigne | *B. p.* | P | n. a. |  |  |  |  |  |  |  |
| Rou5 | Roucadour, France | L-ROU/05 | Middle  Neolithic, Chasséen C2a | J-D Vigne | *B. p.* | P | n. a. |  |  |  |  |  |  |  |
| Rou6 | Roucadour, France | L-ROU/06 | Middle  Neolithic, Chasséen C2a | J-D Vigne | *B. p.* | P | M | A/2 | B/1 | C/1 | Y2 | A/2 | B/2 |  |
|  |  |  |  |  |  |  |  | A/2 | B/1 | C/1 |  | A/1 | B/1 | C/1 |
| Rou8 | Roucadour, France | L-ROU/08 | Final Neolithic C4 | J-D Vigne | *B. p.* | P | n. a. |  |  |  |  |  |  |  |
| Rou9 | Roucadour, France | L-ROU/09 | Final Neolithic final C4 | J-D Vigne | *B. p.* | P | n. a. |  |  |  |  |  |  |  |
| Svo1 | Svodin, Slovakia | 1159 SBSK 4103.49 | 3000 BC, Lengyel | H-P Uerpmann | *B. t.* | T3 | M | A/2 | B/1 | C/1 | Y2 | A/2 | B/1 | C/1 |
|  |  |  |  |  |  |  |  | A/2 | B/2 |  |  | A/2 | B/2 |  |
| Svo3 | Svodin, Slovakia | SVBA 0625/56 | 3000 BC, Lengyel | H-P Uerpmann | *B. t.* | P | M | A/2 | B/2 |  | Y2 | A/2 | B/1 | C/1 |
|  |  |  |  |  |  |  |  | A/2 | B/1 | C/1 |  | A/2 | B/1 | C/1 |
| Tai1 | Taï, France | V-TAI05 Bos1 | Epicardial | J-D Vigne | *B. t.* | T3 | (F) | A/1 | B/1 |  |  |  |  |  |
| Tb22 | Tell Brak, Syra | TB96 A215:2/HN | Chalcolithic | C Edwards, K Dobney | *B. i.­­­­­* | T3 | (F) | A/2 |  |  |  |  |  |  |
| Tre3 | Trebur, Germany | LfD AD EV 1988: 79 Grave 113 | Middle Neolithic | H Göldner | *B. t.* | T3 | (F) | A/1 | B/1 |  |  |  |  |  |
| Tre4 | Trebur, Germany | LfD AD EV 1988:79 Grave 63 | Middle Neolithic | H Göldner | *B. t.* | T3 | F | A/2 | B/1 |  |  |  |  |  |
| Wan4 | Wangels, Germany | Wa 505-97/48 | Early Neolithic | S Hartz, U Schmölke | *B. t.* | T3 | n. a. |  |  |  |  |  |  |  |
| Wan5 | Wangels, Germany | WA 505-98/390 | Early Neolithic | S Hartz, U Schmölke | *B. t.* | T3 | F | A/2 | B/1 |  |  |  |  |  |
| Wan7 | Wangels, Germany | Wa 505-97/105 | Early Neolithic | S Hartz, U Schmölke | *B. t.* | T3 | F | A/2 | B/2 |  |  |  |  |  |
| Wan9 | Wangels, Germany | Wa 505-97/262 | Early Neolithic | S Hartz, U Schmölke | *B. t.* | T3 |  |  |  |  |  |  |  |  |
| Wan10 | Wangels, Germany | Wa 505-97/182 | Early Neolithic | S Hartz, U Schmölke | *B. t.* | T3 | F | A/2 | B/2 |  |  |  |  |  |

(Please note: Accession numbers for GenBank are on the way!)
