# Supplementary material for: Y-SNPs Do Not Indicate Hybridisation between European Aurochs and Domestic Cattle
Source: PLoS One. 2008 Oct 14;3(10):e3418. doi: 10.1371/journal.pone.0003418 (PMC2561061; doi:10.1371/journal.pone.0003418)
Supplement: Table S2 — Primers (0.04 MB DOC) [file pone.0003418.s002.doc]

Table S2: Primers used in this study. U and l mark the upper and lower primer, respectively. Positions according to Genbank accessions: *NM_177490; **NM_177491; ***AY936543; ****AF241271.

|  | Sequence (5´-3´) | Position of amplicon (excluding Primer) | Length with / without Primer (bp) |
| --- | --- | --- | --- |
| *Zinkfinger:* |  |  |  |
| ZFX 3 u | agt gag tcc ata cac gtg tct gac a | 459-486* | 76/27 |
| ZFX 3 l | cga ttt ctg cct cta cta cgc tat |
| ZFY 3 u | gtc ttg acc agt gag tct gta cat | 424-450** | 76/27 |
| ZFY 3 l | cct cta cta cac tac cat gaa caa t |
| *UTY19:* |  |  |  |
| YSNP1u | acg ttc aaa gtt gtt tac aaa aat tc | 403-440*** | 86/37 |
| YSNP 1 l | gaa agt ttg acc ctc ata tgg ct |
| *ZFYintron5:* |  |  |  |
| YSNP 2 u | agt ttt ctg gac caa att cac ttt at | 685-732**** | 102/48 |
| YSNP 2 l | aag tga gtt ctg aac aca ttt aca agt a |
