# Supplementary material for: Y-SNPs Do Not Indicate Hybridisation between European Aurochs and Domestic Cattle
Source: PLoS One. 2008 Oct 14;3(10):e3418. doi: 10.1371/journal.pone.0003418 (PMC2561061; doi:10.1371/journal.pone.0003418)
Supplement: Table S3 — PCR protocol (0.03 MB DOC) [file pone.0003418.s003.doc]

Table S3: PCR reagents and their concentrations. Denaturation: 40 sec 94°C, Hybridisation: 40 sec 52°C (Y-SNP primer) 56°C (ZFX/Y primer), Elongation : 40 sec 72 °C, final elongation 30 min 60°C

| Reagent | Concentration  regular PCR (45 cycles) | extra sensitive PCR (50 cycles) |
| --- | --- | --- |
| 10x PCR Gold Buffer II | 1x; 15 mM Tris HCl, pH 8, 50 mM KCl | 1x; 15 mM Tris HCl, pH 8, 50 mM KCl |
| dNTP Mix | 2,5 M | 2,5 M |
| MgCl_2_ | 0,25 mM | 0,2 mM |
| AmpliTaq Gold™ | 2,5 U | 3,5 U |
| Primer (10µM), each | 200 mM | 200 mM |
| BSA | 20 mg/ml | 20 mg/ml |
| HPLC-H_2_O | Fill up to 50 µl volume | Fill up to 50 µl volume |
| *Target* | 6-10 µl | 6-10 µl |
