# Supplementary material for: Y-SNPs Do Not Indicate Hybridisation between European Aurochs and Domestic Cattle
Source: PLoS One. 2008 Oct 14;3(10):e3418. doi: 10.1371/journal.pone.0003418 (PMC2561061; doi:10.1371/journal.pone.0003418)
Supplement: Table S4 — GenBank accession numbers of sequences presented in this study. (0.04 MB DOC) [file pone.0003418.s004.doc]

Table S4: GenBank accession numbers of sequences presented in this study.

| Probe | HVR1 | ZFX | ZFY | UTY19 | ZFY5 indel |
| --- | --- | --- | --- | --- | --- |
| ALB3 | DQ915520 | FJ005292 | FJ005309 | FJ005266 | FJ005279 |
| ALB4 | DQ915521 | FJ005293 | FJ005310 | FJ005267 | FJ005280 |
| AP7 | FJ005305 | FJ005294 | FJ005311 | FJ005268 | FJ005281 |
| CAT1 | DQ915523 | FJ005295 | FJ005312 | FJ005269 | FJ005282 |
| HAL1 | DQ915552 | FJ005296 | FJ005313 | FJ005270 | FJ005283 |
| PAR1 | DQ915560 | FJ005297 | FJ005314 | FJ005271 | FJ005284 |
| POL5 | FJ005306 | FJ005298 | FJ005315 | FJ005272 | FJ005285 |
| ROS3 | DQ915563 | FJ005299 | FJ005316 | FJ005273 | FJ005286 |
| ROS5 | DQ915565 | FJ005300 | FJ005317 | FJ005274 | FJ005287 |
| ROS7 | DQ915566 | FJ005301 | FJ005318 | FJ005275 | FJ005288 |
| ROU6 | FJ005307 | FJ005302 | FJ005319 | FJ005276 | FJ005289 |
| SVO1 | FJ005308 | FJ005303 | FJ005320 | FJ005277 | FJ005290 |
| SVO3 | DQ915571 | FJ005304 | FJ005321 | FJ005278 | FJ005291 |
